# Palindromic Minocycline-Induced Vasculitis With Dual-Positivity for Myeloperoxidase Anti-neutrophil Cytoplasmic Antibody (MPO-ANCA) and Anti-DNA Antibody

**DOI:** 10.7759/cureus.92030

**Published:** 2025-09-11

**Authors:** Hiroto Tomoda, Yoshitaka Ueda

**Affiliations:** 1 Department of Rheumatic Diseases, Tokyo Metropolitan Tama Medical Center, Tokyo, JPN

**Keywords:** anca-associated vasculitis, drug-induced vasculitis, minocycline, mpo-anca, vasculitis

## Abstract

Long-term usage of minocycline can result in the development of various autoimmune conditions, including vasculitis. We herein report the case of a 23-year-old female patient who developed fever, arthralgia, and erythema of the lower limbs 15 months after the initiation of minocycline. Her symptoms recovered spontaneously within one and a half months but relapsed two months later along with dysesthesia of the lower limbs. Her myeloperoxidase anti-neutrophil cytoplasmic antibody (MPO-ANCA) and anti-DNA antibody were positive, and histology of her skin demonstrated features of necrotizing vasculitis of the small vessels. Minocycline was discontinued, and her symptoms improved without requiring further treatment, along with the normalization of MPO-ANCA and anti-DNA antibody titers. No recurrence was observed nine months after discontinuation, although her lower limb dysesthesia lasted for six months before recovery. Clinicians should bear in mind the possibility of minocycline-induced vasculitis when symptoms of vasculitis are suspected in patients receiving long-term minocycline. An immediate withdrawal of the drug is recommended in such cases to prevent the development of organ-threatening lesions and long-lasting aftereffects.

## Introduction

Drug-induced vasculitis is a type of secondary vasculitis triggered by an adverse reaction to certain medications and is often associated with the presence of anti-neutrophil cytoplasmic antibodies (ANCA) [1]. Symptoms include rash, fever, arthralgia, myalgia, and in severe cases, neuropathic, renal, or pulmonary involvement. Several therapeutic agents have been reported to cause this phenomenon, including antibiotics, antithyroid medications, and anticonvulsants. Treatment typically involves discontinuation of the suspected drug, and in some cases, corticosteroids or other immunosuppressive therapy for severe organ manifestations.

Minocycline is a broad-spectrum tetracycline antibiotic used to treat various bacterial infections, especially acne vulgaris. While generally well tolerated, it can cause a wide variety of autoimmune adverse effects, including drug-induced lupus, autoimmune hepatitis, and vasculitis [2]. Minocycline-induced vasculitis is a rare complication associated with prolonged minocycline therapy, presumed to be less frequent compared to lupus or hepatitis [2,3]. Only sporadic case reports and case series have been documented thus far [3-24]. It commonly presents with polyarteritis nodosa (PAN)-like features and is more frequently observed in younger patients compared to other drug-induced vasculitides, presumably because it often develops in patients treated for acne [4]. It is often accompanied by myeloperoxidase ANCA (MPO-ANCA) and antinuclear antibody (ANA) positivity; however, anti-DNA antibody positivity is rarely documented, with only one case described to date [3,4]. We report a case of palindromic minocycline-induced vasculitis with an overlap of MPO-ANCA, ANA, and anti-DNA antibodies.

## Case presentation

**Figure 1:**
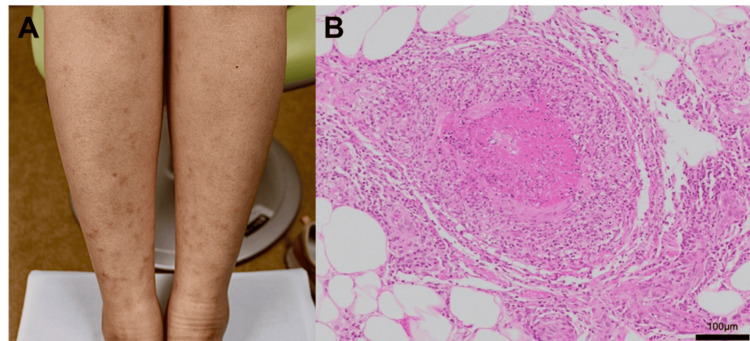
Clinical features of minocycline-induced vasculitis (A) Multiple indurated blanching erythemas are shown in a photograph of the patient’s legs, but no purpura is observed. (B) 200x hematoxylin and eosin staining of her left leg skin reveals an infiltration of inflammatory cells, especially lymphocytes and neutrophils, in the small vessel (100-190 μm in diameter) walls and the surrounding tissue, with fibrinoid necrosis of the vessel walls.

A 23-year-old Japanese female presented to our rheumatology department with fever, arthralgia, and erythema of the lower limbs, which developed one and a half months prior to her visit (Figure 1A). Her blood pressure was 112/60 mmHg. She had been taking minocycline for acne treatment for 15 months. Her symptoms resolved without treatment, and a viral infection was suspected as the cause. Two months later, however, she revisited our hospital complaining of dysesthesia and numbness in her feet, in addition to the recurrence of her previous symptoms. Sensory impairment was observed in areas innervated by the peroneal and sural nerves, and nerve conduction studies revealed a reduced sensory action potential of the sural nerve. No muscle weakness was noted. The C-reactive protein (CRP) value increased to 1.93 mg/dL from 0.06 mg/dL at the time of her first visit. MPO-ANCA was 17.9 U/mL (< 3.5 U/mL), ANA titer was 1:40 (homogenous and speckled pattern), and anti-DNA antibody was 7.3 IU/mL (< 6.0 IU/mL) (see Table 1 for additional laboratory results). She was admitted to our hospital, and skin biopsy findings of her left lower leg showed perivascular inflammatory cell infiltration and fibrinoid necrosis of the small vessel walls (Figure 1B), which were consistent with necrotizing vasculitis. No lung involvements or other findings suggestive of vasculitis, including aneurysms, stenosis, and vessel wall thickening, were found in a computed tomography scan of the body. No signs of nephritis were noted in a blood and urine test, and no intestinal involvements were found in an upper and lower gastrointestinal endoscopy. The patient was negative for hepatitis B and C, cytomegalovirus, and Epstein-Barr virus. The palindromic and long-lasting course of her symptoms made the possibility of other viral infections unlikely. She did not have a family history of vasculitis or other rheumatic diseases, nor did she have a history of exposure to toxins or drugs other than minocycline. The low titers of ANA along with normal complements and rheumatoid factor made the diagnosis of systemic lupus erythematosus or rheumatoid vasculitis unlikely.

Minocycline-induced vasculitis was suspected, and the medication was discontinued. Her fever, arthralgia, and erythema improved, and the CRP value returned to normal (0.05 mg/dL) without requiring further treatment. She was discharged from the hospital 10 days after admission. The MPO-ANCA titer decreased to 5.1 U/mL and 2.5 U/mL one and four months after the withdrawal of minocycline, respectively. Her anti-DNA antibody titer also normalized to 5.0 IU/mL one month after withdrawal. Nine months after discontinuing minocycline, the patient is currently receiving no medication for her vasculitis and has not experienced a recurrence to date. However, dysesthesia of her feet lasted for six months before recovery.

**Table 1:** Laboratory data at the time of admission MPO: myeloperoxidase, ANCA: anti-neutrophil cytoplasmic antibody, DNA: deoxyribonucleic acid * Anti-single-strand DNA antibody, perinuclear-ANCA, and cytoplasmic-ANCA were not measured in this patient.

Variable	On admission	1 month after minocycline withdrawal	Reference range
Erythrocyte sedimentation rate (mm/hr)	29	N/A	<15
C‐reactive protein (mg/dL)	1.93	0.01	<0.14
Antinuclear antibody	(+) 1:40, homogenous, speckled	N/A	<1:40
Anti-DNA antibody (IU/mL)	7.3	5	<6.0
Anti-double-strand DNA antibody (IU/mL)	<10	N/A	<12
Anti-ribonucleoprotein antibody (U/mL)	<2.0	N/A	<10.0
Anti-Smith antibody (U/mL)	<1.0	N/A	<10.0
MPO-ANCA (U/mL)	17.9	5.1	<3.5
Proteinase 3-ANCA (U/mL)	<1.0	N/A	<2.0
Rheumatoid factor (IU/mL)	5	N/A	<15
Immunoglobulin G (mg/dL)	2,218	N/A	861-1,747
Immunoglobulin A (mg/dL)	199	N/A	93-393
Immunoglobulin M (mg/dL)	81	N/A	50-269
C3 (mg/dL)	138	107	73-138
C4 (mg/dL)	24.9	15	11.0-31.0
Hepatitis B surface antigen	Negative	N/A	Negative
Anti-hepatitis C antibody	Negative	N/A	Negative
White blood cell count (per μL)	5,600	5,300	3,300-8,600
Hemoglobin (g/dL)	10.6	10.8	11.6-14.8
Platelet count (per μL)	353,000	269,000	158,000-348,000
Albumin (g/dL)	4.0	4.5	4.1-5.1
Aspartate aminotransferase (U/L)	32	29	13-30
Alanine aminotransferase (U/L)	38	40	7-23
Creatinine kinase (U/L)	25	42	41-153
Creatinine (mg/dL)	0.47	0.42	0.46-0.79
Urine protein	Negative	Negative	Negative
Urine blood	Negative	Negative	Negative

## Discussion

Minocycline-induced vasculitis is a rare complication in patients who are receiving long-term doses of the drug. In a case series of nine patients, the mean duration from minocycline administration to the development of vasculitis was 2.1 years [4]. Most patients, including our case, develop vasculitis after at least one year of minocycline use, though a few rare exceptions have been reported [5,6]. Elkayam et al. have reported that the same applies to other minocycline-induced symptoms, including drug-induced lupus and autoimmune hepatitis [2]. Except for serum sickness, which occurred after a mean of 16 days, the mean time from initiation of minocycline to the development of autoimmune conditions (vasculitis, drug-induced lupus, and autoimmune hepatitis) was 25.3 months.

Culver et al. proposed a diagnostic guideline for minocycline-induced vasculitis, in which this condition is considered when a patient meets at least six of the following seven criteria: (1) minocycline use for >12 months, (2) skin manifestations including livedo reticularis and/or subcutaneous nodules, (3) arthritis and/or myalgias and/or neuropathy in the distribution of the rash, (4) lack of systemic organ involvement, (5) skin biopsy with necrotizing vasculitis of small- and/or medium-sized vessels, (6) the presence of perinuclear-ANCA (p-ANCA), and (7) improvement after discontinuation of minocycline [7]. All seven criteria are satisfied in our case (although p-ANCA is substituted for MPO-ANCA in our case), making our diagnosis of minocycline-induced vasculitis likely. Because our case was positive for anti-DNA antibodies, it was challenging to distinguish minocycline-induced vasculitis from minocycline-induced lupus. However, the presence of MPO-ANCA, the symptoms of multiple mononeuropathy, and the histological findings of necrotizing vasculitis support the diagnosis of vasculitis rather than lupus.

It has been reported that most cases of minocycline-induced vasculitis are p-ANCA or MPO-ANCA positive, and some are ANA positive [1,3,4]. In a case series and review of literature by Kermani et al., 15 out of the 19 cases in which p-ANCA was tested were positive for p-ANCA [4]. MPO-ANCA was positive in only four of these patients, although MPO was not measured in some patients. ANA was positive in 10 out of the 17 patients tested, but anti-DNA antibody positivity has been reported in only one case. To our knowledge, anti-DNA antibodies were not detected in any of the previous reports of minocycline-induced vasculitis, except for this case. By contrast, Elkayam et al. have reported anti-DNA antibody positivity in four out of 35 cases of minocycline-induced lupus and in seven out of 22 cases of autoimmune hepatitis. However, none of the four cases of vasculitis had anti-DNA antibodies [2].

The anti-DNA antibody and ANA titers in our case were relatively mild, and the possibility of nonspecific elevation cannot be excluded. However, the ANA patterns reported in previous studies were also mostly mild with an ANA of 1:40 to 1:160 in many cases [3,7-12], except for two cases with an ANA of 1:320 [5,13], four with 1:640 [14-16], and one with 1:1280 [17]. The ANA patterns were also diverse, with cases of homogenous [3,8,10,11], speckled [7,10,13,15], and nucleolar [8,14,16] patterns all being reported. A case with anti-centromere and anti-single-strand DNA antibody has also been described previously [5]. From these experiences, it can be inferred that autoantibodies detected in minocycline-induced vasculitis are typically mild but exhibit diverse variations.

The mechanism of anti-DNA antibody production in our case remains largely unknown. Previous studies in the field of drug-induced lupus, however, hypothesize that the generation of autoantibodies against nucleosome subunits, especially the (H2A-H2B)-DNA complex, is the mechanism underlying anti-DNA antibody positivity [25]. Further studies are needed to determine whether a similar mechanism is responsible for the production of autoantibodies in minocycline-induced vasculitis.

Sporadic cases of minocycline-induced vasculitis in Japanese patients have been reported, with at least six cases being published to date [5,8,10,11,13]. Kawahara et al. have proposed that a specific type of human leukocyte antigen (HLA), notably HLA-DRB1*09:01, which is more common in Asian populations compared to European patients, may play a role in the pathogenesis of minocycline-induced vasculitis. HLA was not tested in our patient, however. Similar to our case, two of these cases presented with neuropathy [5,10], three were ANCA positive [8,11], and four were ANA positive [5,8,11,13]. Accumulation of additional reports is necessary to determine if there is a correlation between ethnicity and disease prevalence or phenotype.

The symptoms of minocycline-induced vasculitis are usually mild, but neuropathy is reported in some cases [4-6,9,10,18-20]. Other severe organ manifestations previously reported include brainstem stroke [21], myelopathy [22], renal PAN (including renal hypertension, aneurysms, and infarctions) [12,13], and testicular infarction [23]. Minocycline withdrawal is usually effective, but prednisolone and other immunosuppressive therapies may be considered in cases with severe neuropathy and other organ-threatening lesions. Reinitiation of minocycline is not recommended, even after remission is achieved, as recurrence of previous symptoms has been reported in cases where minocycline was rechallenged [10,20,24]. In this present case, symptoms of minocycline-induced vasculitis spontaneously improved without cessation of the drug but reappeared with increasing severity. Based on these experiences, it is recommended that minocycline be withdrawn immediately when suspected symptoms of vasculitis appear and that it should not be rechallenged even after remission has been achieved.

## Conclusions

This case has demonstrated that clinicians should be vigilant of vasculitis and other autoimmune conditions when administering long-term doses of minocycline. This is the second report of minocycline-induced vasculitis with anti-DNA positivity. Anti-DNA antibodies, although less common than p-ANCA or ANA, may be present in some cases of minocycline-induced vasculitis, making it challenging to distinguish from drug-induced lupus. Symptoms of minocycline-induced vasculitis may recur and leave long-lasting neurologic aftereffects, so minocycline should be discontinued immediately when symptoms of vasculitis are suspected in patients receiving the drug.
